# Supplementary material for: Optimal Representative Strain selector—a comprehensive pipeline for selecting next-generation reference strains of bacterial species
Source: NAR Genom Bioinform. 2024 Dec 18;6(4):lqae173. doi: 10.1093/nargab/lqae173 (PMC11655286; doi:10.1093/nargab/lqae173)
Supplement: lqae173_Supplemental_Files [file lqae173_supplemental_files.zip › SUPPLEMENTARY FIGURE LEGENDS.docx]

**SUPPLEMENTARY FIGURE LEGENDS**

**Figure S1. Phylogenetic investigation of *Streptococcus thermophilus* and *Lactobacillus delbrueckii* species.** Panel a) shows the core gene-based phylogenetic tree illustrating the relationships among the 91 non-redundant *S. thermophilus* strains processed using the ORS selector software. Panel b) presents the core gene-based phylogenetic tree depicting the evolutionary relationships among the 72 non-redundant *L. delbrueckii* strains. The Type Strain of the species, (or a genomically identical alternative with >99% similarity) is reported in yellow, while the optimal representative species defined by the ORS selector pipeline is reported in violet. The genomic, functional, ecological, and final representative scores for each strain are reported as colored bars.

**Figure S2. Phylogenetic investigation of *Lacticaseibacillus paracasei* species**. The core gene-based phylogenetic tree illustrates the relationships among the 74 non-redundant *L. paracasei* strains processed using the ORS selector software. Type Strain of the species, (or a genomically identical alternative with >99% similarity) is reported in yellow, while the optimal representative species defined by the ORS selector pipeline is reported in violet The genomic, functional, ecological, and final representative scores for each strain are reported as colored bars.

**Figure S3. Phylogenetic investigation of *Lactococcus lactis* species**. The core gene-based phylogenetic tree illustrates the relationships among the 84 non-redundant *L. lactis* strains processed using the ORS selector software. Type Strain of the species, (or a genomically identical alternative with >99% similarity) is reported in yellow while the optimal representative species defined by the ORS selector pipeline is reported in violet. The genomic, functional, ecological, and final representative scores for each strain are reported as colored bars.

**Figure S4. Phylogenetic investigation of *Bacteroides thetaiotaomicron* species**. The core gene-based phylogenetic tree illustrates the relationships among the 91 non-redundant *B. thetaiotaomicron* strains processed using the ORS selector software. Type Strain of the species, (or a genomically identical alternative with >99% similarity) is reported in yellow, while the optimal representative species defined by the ORS selector pipeline is reported in violet. The genomic, functional, ecological, and final representative scores for each strain are reported as colored bars.
