# Supplementary material for: Optimal Representative Strain selector—a comprehensive pipeline for selecting next-generation reference strains of bacterial species
Source: NAR Genom Bioinform. 2024 Dec 18;6(4):lqae173. doi: 10.1093/nargab/lqae173 (PMC11655286; doi:10.1093/nargab/lqae173)
Supplement: lqae173_Supplemental_Files [file lqae173_supplemental_files.zip › Supplementary_Figures.pdf]

a) **Core gene-based phylogenetic tree of 32 non-redundant *Streptococcus thermophilus* genomes**

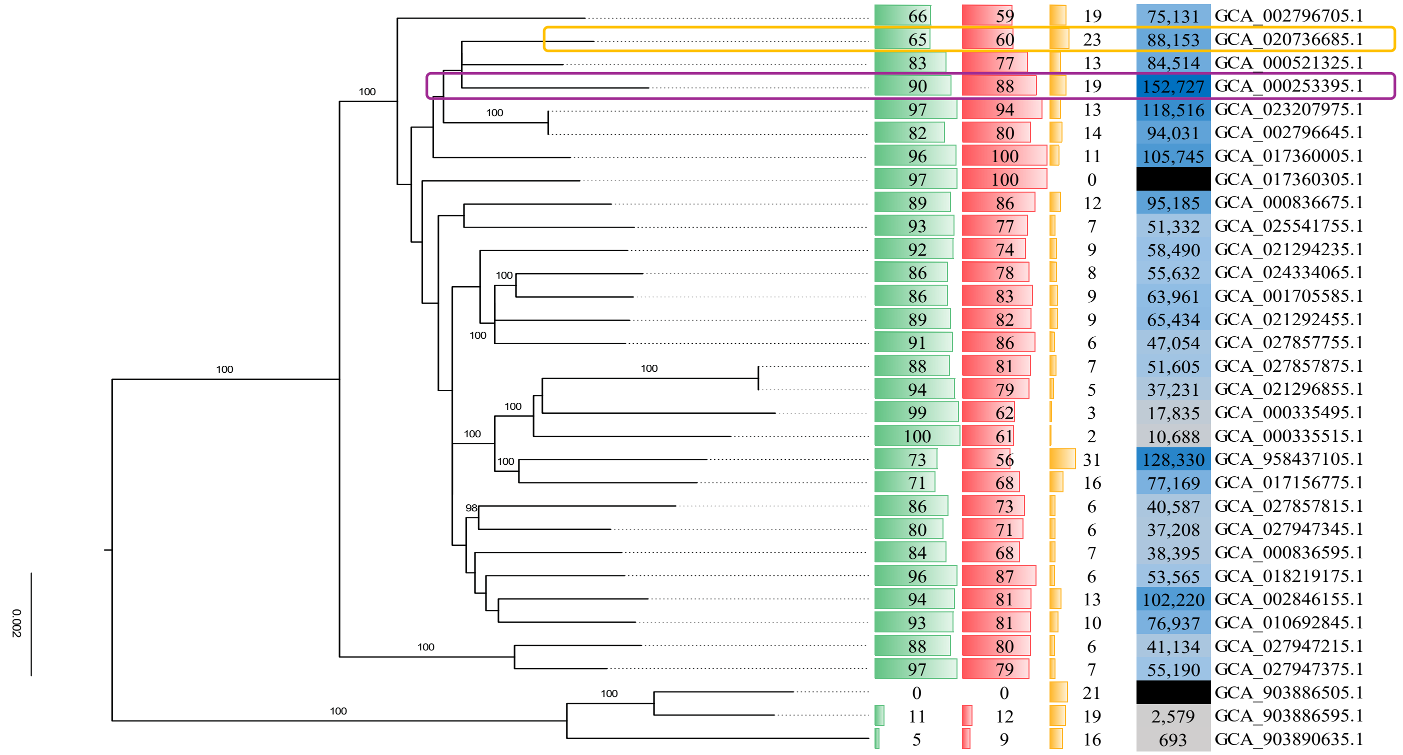

b) **Core gene-based phylogenetic tree of 72 non-redundant *Lactobacillus delbrueckii* genomes**

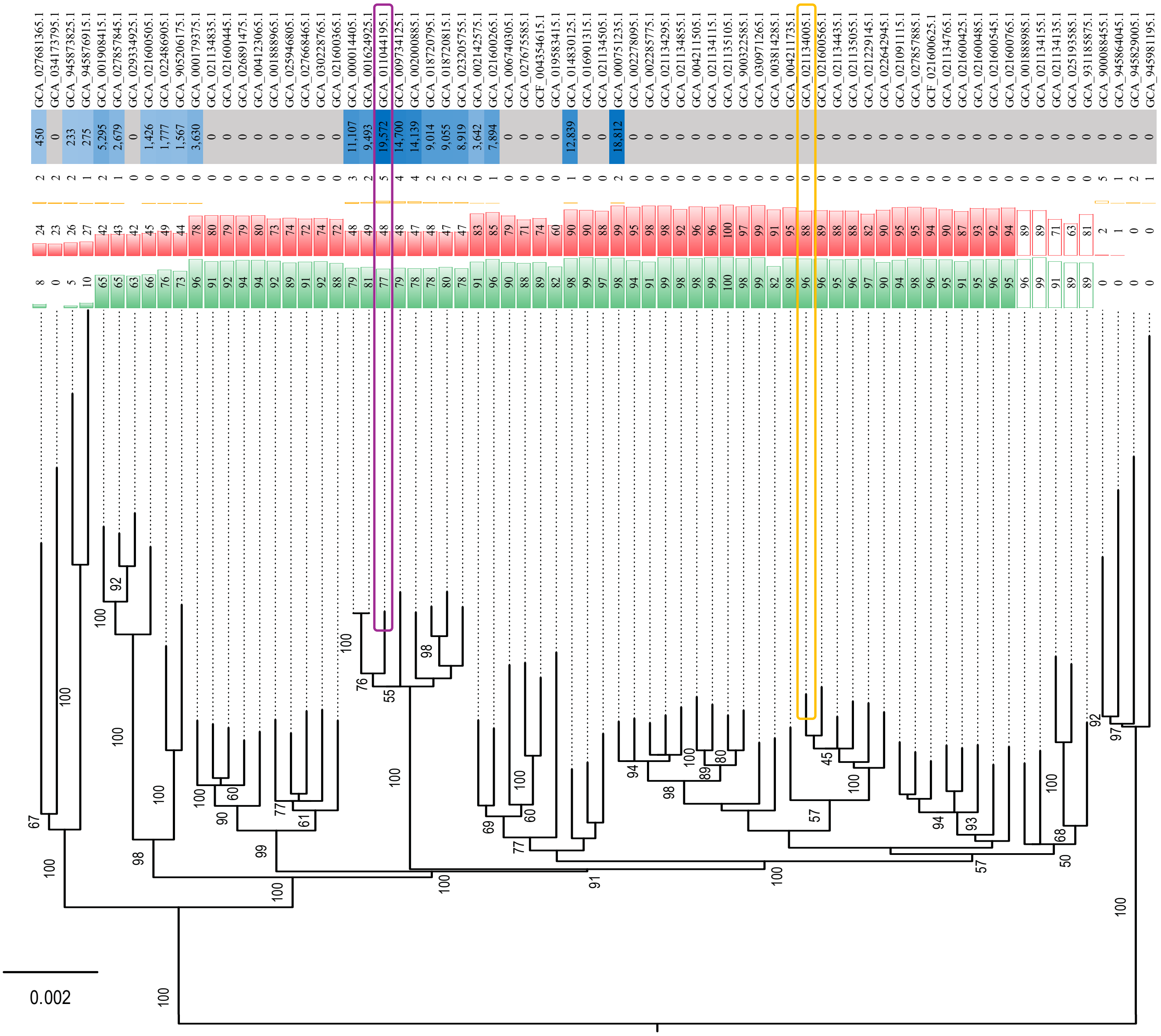

Figure S1

Core gene-based phylogenetic tree of 74 non-redundant *Lacticaseibacillus paracasei* genomes

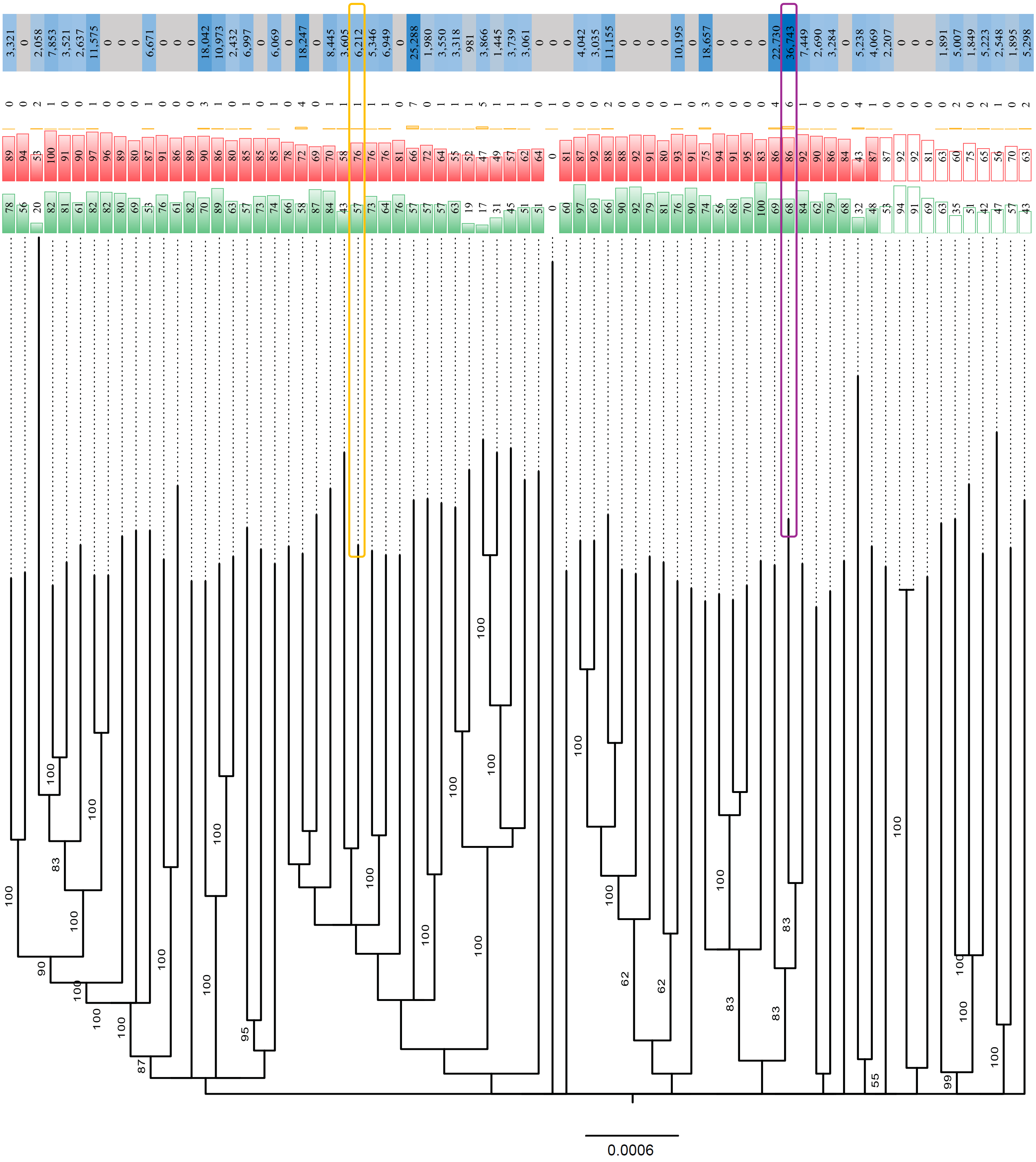

Figure S2

Core gene-based phylogenetic tree of 84 non-redundant *Lactococcus lactis* genomes

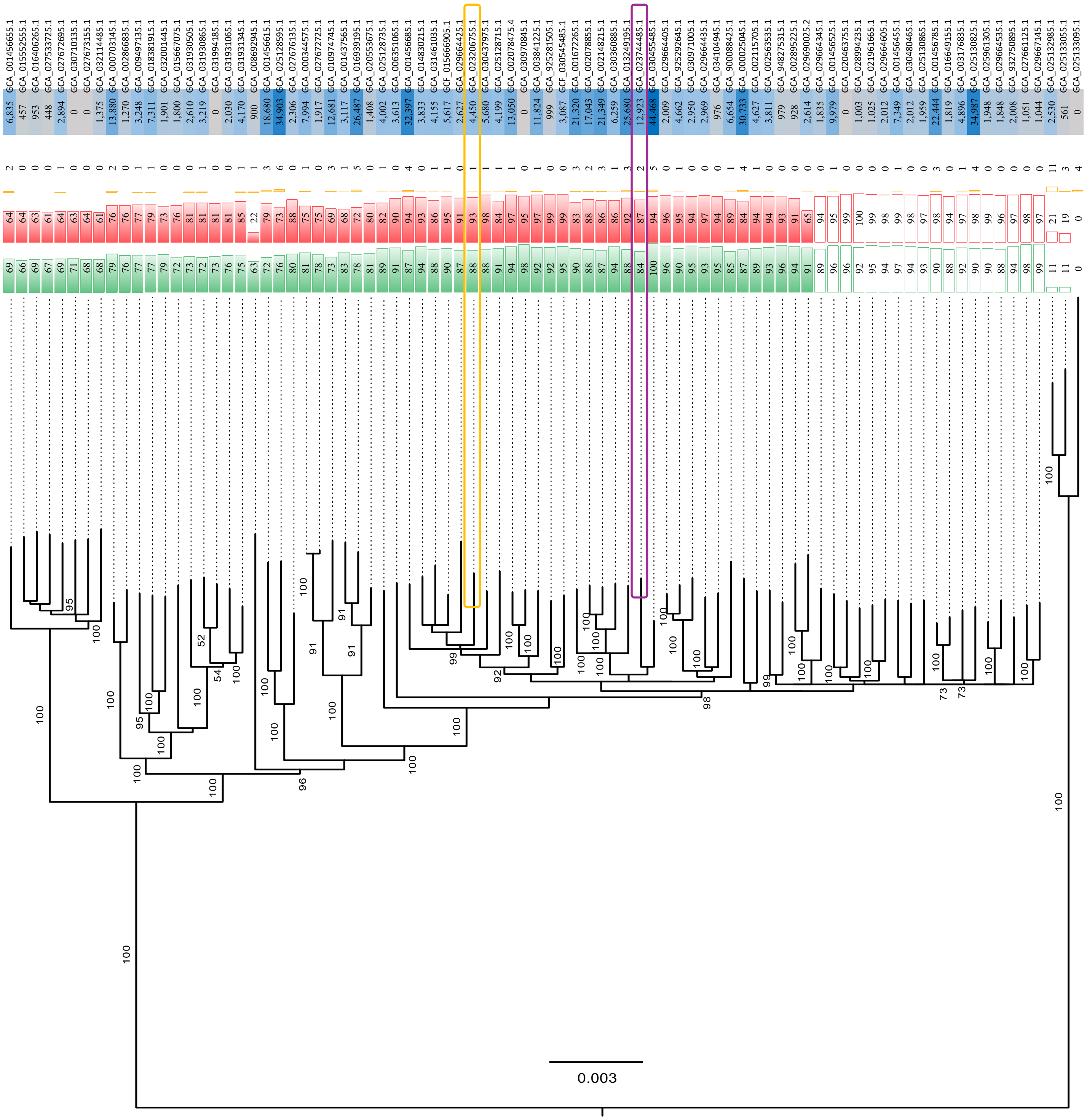

Figure S3

Core gene-based phylogenetic tree of 91 non-redundant *Bacteroides thetaiotaomicron* genomes

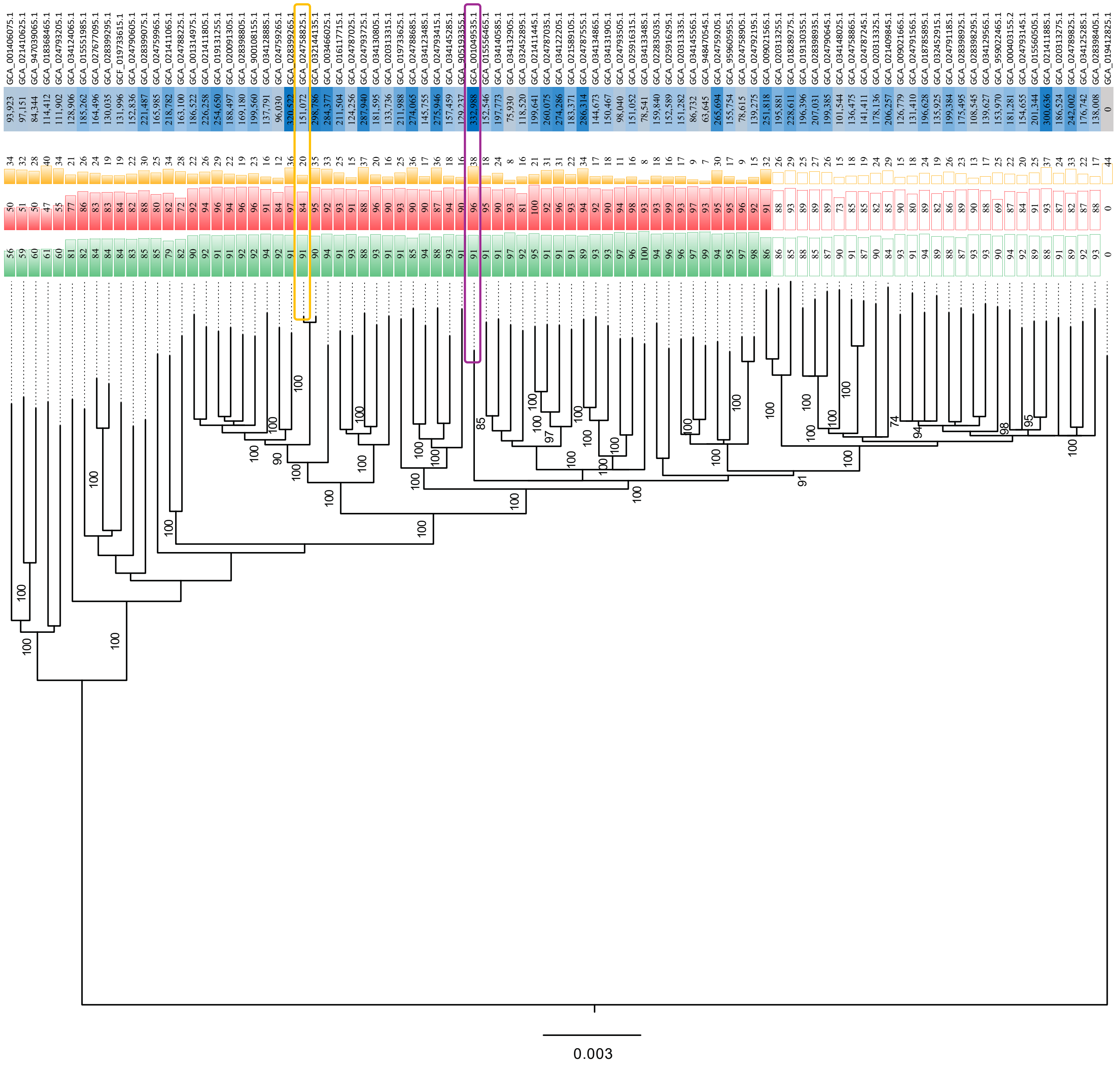

Figure S4
